# Star polyester-based folate acid-targeting nanoparticles for doxorubicin and curcumin co-delivery to combat multidrug-resistant breast cancer

**DOI:** 10.1080/10717544.2021.1960926

**Published:** 2021-08-31

**Authors:** Fangyuan Guo, Nan Yu, Yunlong Jiao, Weiyong Hong, Kang Zhou, Xugang Ji, Huixing Yuan, Haiying Wang, Aiqin Li, Guoping Wang, Gensheng Yang

**Affiliations:** a College of Pharmaceutical Science, Zhejiang University of Technology, Hangzhou, China; b Department of Pharmacy, Taizhou Municipal Hospital of Zhejiang Province, Taizhou, China; c Zhejiang Share Bio-pharm Co., Ltd, Hangzhou, China; d Zhejiang Dayang Biotech Group Co., Ltd, Jiande, China

**Keywords:** Folate acid-targeting nanoparticles, combination therapy, multidrug-resistance, breast cancer

## Abstract

Chemotherapeutic treatments are indispensable in the treatment of breast cancer. However, the emergence of multidrug-resistance, strong cell toxicity, and poor targeting selection has inhibited their clinical application. In this study, two synergistic drugs, doxorubicin (DOX) and curcumin (CUR), were co-administered to overcome multidrug resistance (MDR). Based on the characteristics of the tumor microenvironment, we developed folic acid-modified nanoparticles ((DOX + CUR)-FA-NPs) based on a star-shaped polyester (FA-TRI-CL) to enhance the tumor targeting selectivity and drug loading (DL) capacity. The (DOX + CUR)-FA-NPs displayed a characteristic spheroid morphology with an ideal diameter (186.52 nm), polydispersity index (0.024), zeta potential (–18.87 mV), and good entrapment efficiency (97.64%/78.13%, DOX/CUR) and DL (20.27%/11.29%, DOX/CUR) values. *In vitro* pharmacokinetic and pharmacodynamic experiments demonstrated that the (DOX + CUR)-FA-NPs were gradually released, and they displayed the highest cell apoptosis and cellular uptake in MCF-7/ADR cells. Additionally, *in vivo* results illustrated that (DOX + CUR)-FA-NPs not only displayed significant tumor targeting and anticancer efficacy, but also induced less pathological damage to the normal tissue. In summary, co-administered DOX and CUR appeared to reverse MDR, and this targeted combinational nanoscale delivery system could thus be a promising carrier for tumor therapies in the future.

## 1. Introduction

Breast cancer is one of the most common malignancies in women (Siegel et al., 2020), and chemotherapeutic treatments are an indispensable clinical method used in the fight against it. However, the emergence of multidrug resistance (MDR) during treatment has greatly hindered the therapeutic efficacy of chemotherapeutic drugs (Robey et al., 2018; Liu et al., 2019). The main mechanisms of MDR are summarized as follows: high expression of drug-transporting proteins (e.g. P-gp, etc.; Zhao et al., 2018; Hou et al., 2019), activation of cellular anti-apoptotic defense (Kirkin et al., 2004; Xu et al., 2020), and topoisomerase activity reduction (Liu et al., 2020). Recent studies have demonstrated that MDR might facilitate tumor metastasis (Xu et al., 2017) and recurrence (Lang et al., 2019). Therefore, effective strategies to overcome MDR are urgently required.

Co-delivery of anticancer drugs with different antitumor mechanisms is a promising strategy to combat MDR (Li & Xie, 2017; Shi et al., 2021). Doxorubicin (DOX) is one of the most effective drugs for the treatment of breast cancer and functions mainly through DNA insertion and topoisomerase II inhibition to achieve apoptosis and the inhibition of cell growth (Cheung et al., 2009; Zhang et al., 2018). However, the emergence of MDR (by the P-gp pathway; Li et al., 2018), strong cell toxicity, and poor targeting selection have seriously hindered its clinical application. Curcumin (CUR) is a low-toxicity natural drug made of polyphenols extracted from Zingiberaceae plants (Wu et al., 2019) and is less effective against cancer than first-line chemotherapy but has broad-spectrum effects (Guo et al., 2018; Zhang et al., 2018; Barati et al., 2019; Guo et al., 2019, 2020). Hou et al. (2019) and Lv et al. (2016) have shown that CUR is an excellent P-gp inhibitor that effectively maintains a high DOX concentration in drug-resistant human breast cancer MCF-7/ADR cells. Additionally, recently published studies (Mohajeri & Sahebkar, 2018; Barati et al., 2019) showed that CUR has a protective effect on DOX toxicity in the heart, kidney, liver, and blood components. Therefore, the co-delivery of CUR and DOX could be an effective combination oncology therapy but further investigation is required.

The development of nanotechnology means that drug carriers at the nanoscale could simultaneously deliver multiple drugs to tumor sites due to their enhanced permeability and retention effects (EPR), and this could reduce the toxic side effects of drugs and help to regulate drug release (Wang et al., 2014; Vinothini et al., 2019; Ren et al., 2021; Zhang et al., 2021). At present, to improve tumor targeting efficiency, nanoparticles with various targeting ligands that respond to tumor-specific pathological abnormalities (such as low pH, specific receptors, and ROS) have become a new focus of research (Gupta et al., 2019; Hou et al., 2019). The folate receptor, a specific receptor for some cancer cells, including breast cancer cells, with no expression or absence in normal cells, can be used as an active target for breast cancer treatment. Furthermore, published studies that use folic acid (FA) receptors as targets for drug delivery systems with single drug-loads, have achieved effective tumor targeting and prominent anti-cancer efficacy (Anirudhan et al., 2017; Dramou et al., 2018). Low drug loading (DL) capacity, however, is a shortcoming of nanocarriers. For multidrug co-drug deliveries, the above shortcomings will be more pronounced owing to the limitations of the package space inside the nanocarriers. Additionally, the gradient of release between different drugs is another barrier that should be addressed in clinical research. Therefore, new materials that could be used as nanocarriers to better enable high DL and controlled drug release should be developed.

**Figure 1.**
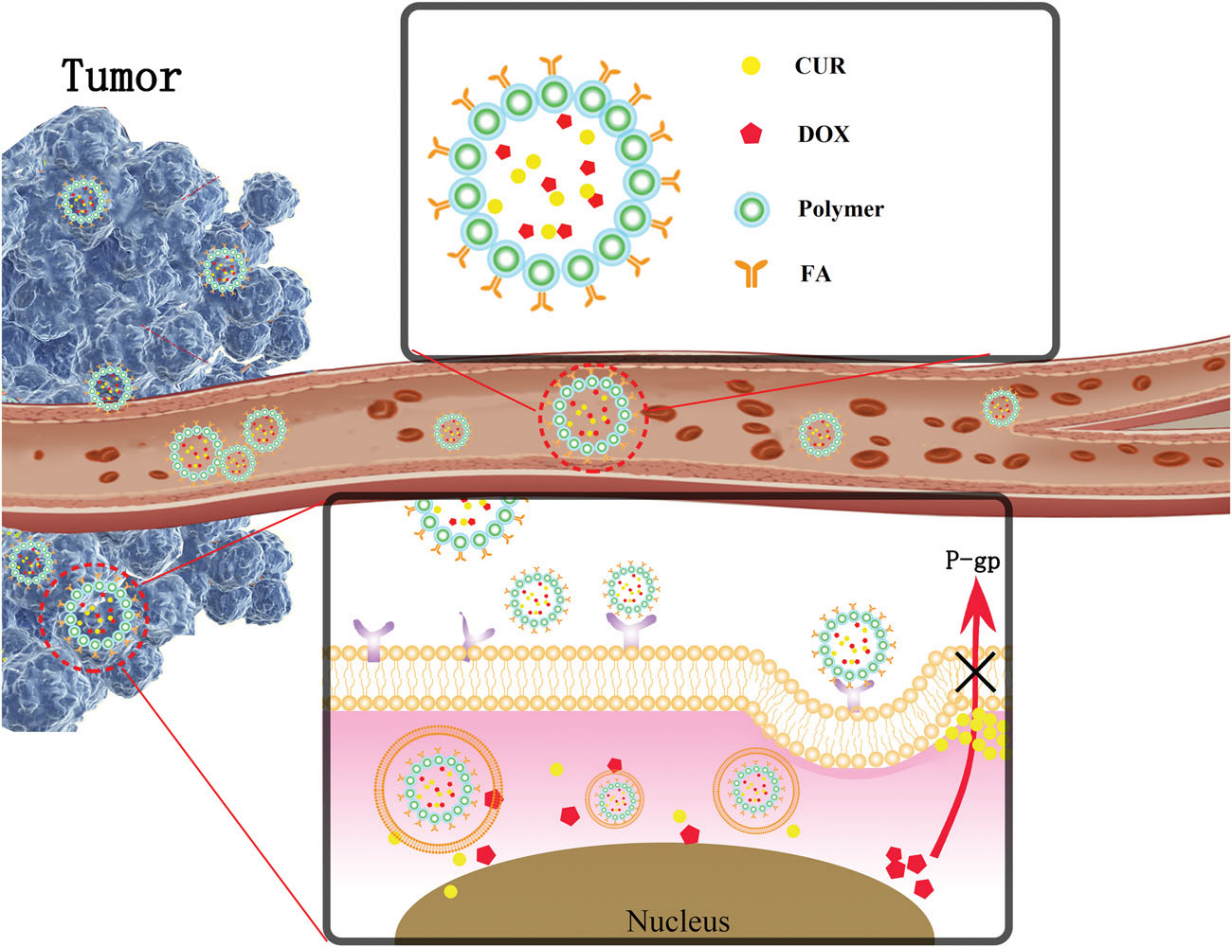
Detailed drug delivery process *in vivo*.

In this study, active tumor-targeting nanoparticles based on the FA-modified star-shaped polymer (aggregation of 1,2,3-propane tricarboxylic acid (TRI) and ε-caprolactone (ε-CL)), which had relatively large internal packaging spaces, were designed and synthesized for the first time. DOX and CUR were the model drugs that were co-delivered inside the nano-delivery system with high DL using the emulsion solvent evaporation method. Thereafter, the security, pharmacokinetics, and pharmacodynamics of these special nanoparticles ((DOX + CUR)-FA-NPs) were evaluated. The detail drug delivery process is displayed in Figure 1. This is expected to be a promising multi-drug co-delivery nano-platform that could reverse MDR and improve anti-cancer efficacy in breast cancer treatments.

## 2. Materials and methods

### 2.1. Materials

CUR, poloxamer 188, stannous 2-ethylhexanoate [Sn(Oct)_2_], and dialysis bags (MWCO = 14,000) were procured from Hangzhou Guang Lin Biological Pharmaceutical Co., Ltd (Hangzhou, China), BASF (Ludwigshafen, Germany), Sigma-Aldrich (St. Louis, MO), and Gene Star Co. (Hangzhou, China), respectively. All test cells were purchased from the Cell Bank of the Chinese Academy of Sciences (Beijing, China). DOX, TRI, ε-CL, and other reagents were procured from Aladdin Chemicals (Shanghai, China).

### 2.2. Synthesis of FA-TRI-PCL

**Figure 2.**
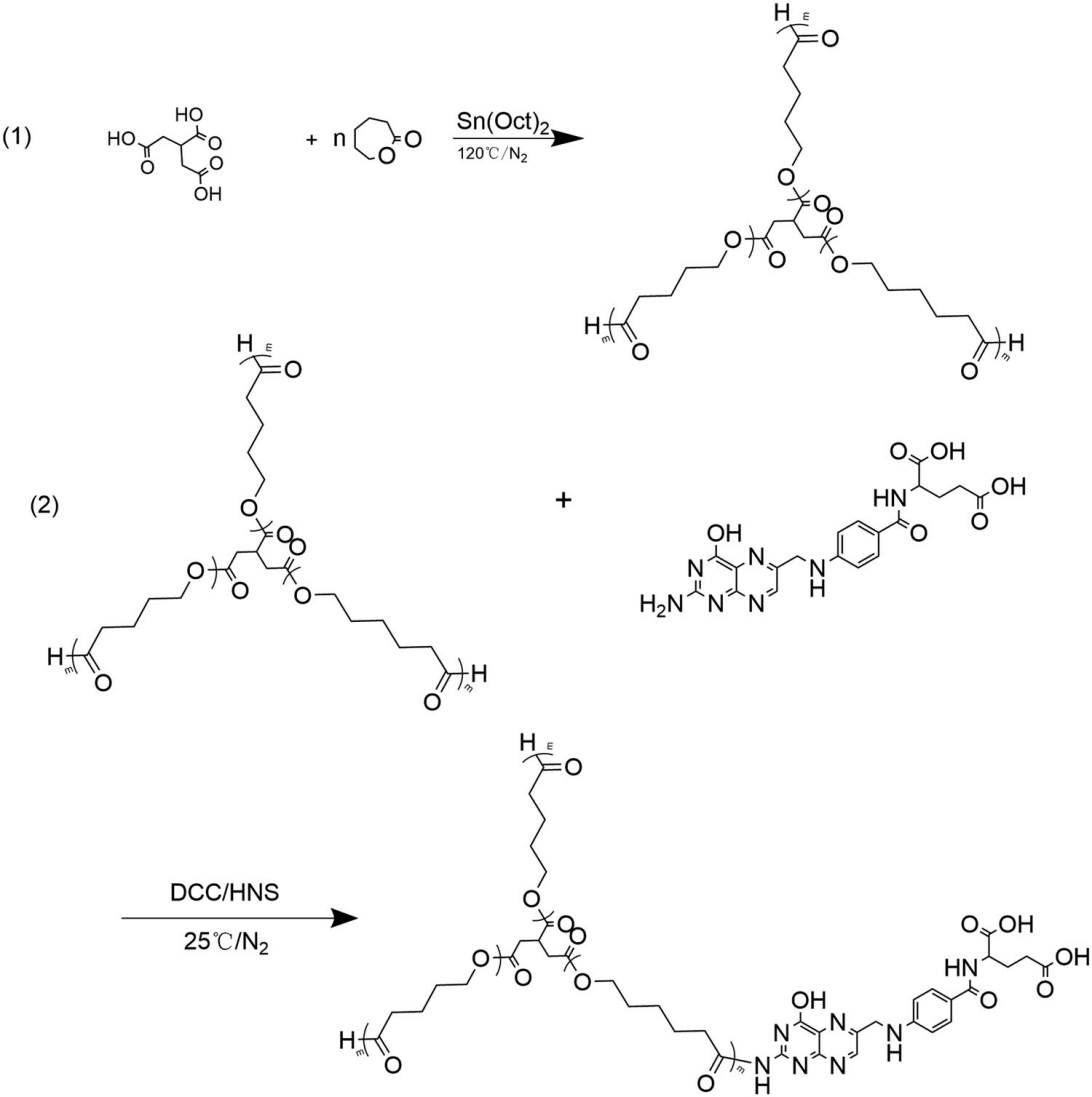
Reaction scheme for FA-TRI-PCL preparation.

FA-TRI-PCL was prepared using ε-CL, tricarballylic acid, and FA. The detailed process is illustrated in Figure 2.

#### 2.2.1. Synthesis of TRI-CL

TRI (1 g), ε-CL (35.8 g), and Sn (Oct)_2_ (catalyst, 0.5% w/w) were mixed and reacted at 120 °C under nitrogen protection. After 24 h of reaction, 25 mL of dichloromethane was added, and the resultant solution was then added dropwise into a 10 times volume of diethyl ether. Thereafter, the white precipitate was collected by filtration. The purification process was repeated twice. The final product (TRI-CL) was dried in a vacuum oven at 25 °C. The molecular structure of the TRI-CL was determined using IR and ^1^H NMR spectroscopy.

#### 2.2.2. Synthesis of FA-TRI-CL

TRI-CL (1000 mg), N,N′-carbonyldiimidazole (CDI, 113.5 mg), and trimethylamine (TEA, 70.8 mg) were mixed, and the reaction was carried out for 6 h in darkness at 25 °C under N_2_ protection. Thereafter, a DMSO solution of FA (309 mg) was added to react for another 24 h at 25 °C. The crude product (FA-TRI-CL) was obtained with the same process used for the TRI-CL purification. The molecular structure of the FA-TRI-CL was determined using IR and ^1^H NMR spectroscopy.

#### 2.2.3. Preparation of (CUR and DOX)-loaded NPs

The (CUR + DOX)-FA-NPs and (CUR + DOX)-NPs were synthesized as follows: CUR (1 mg/mL), DOX (1 mg/mL), and FA-TRI-CL or TRI-CL (15.0 mg/mL) were added drop-wise into a five times volume of aqueous phase containing 0.5% (w/w) Poloxamer-188 under magnetic stirring. To remove the acetone, the stirring process was conducted for another 4 h. The obtained primary (CUR + DOX)-FA-NPs or (CUR + DOX)-NP suspensions were purified by centrifugation at 2817×*g* for 10 min to remove the unwrapped drugs and free FA-TRI-CL or TRI-CL. Additionally, blank FA-NPs or blank NPs were carried out using the same process mentioned above but without the drugs.

#### 2.2.4. Characterization of (CUR + DOX)-loaded NPs

The zeta potential, particle size, and polydispersity index (PDI) were measured using dynamic light scatterometer (DLS). The morphology and drug encapsulation status were analyzed using X-ray powder diffraction (XRD) and transmission electron microscopy (TEM), respectively. For entrapment efficiency (EE) and DL, the analysis process was as follows: the purified (CUR + DOX)-FA-NPs were harvested by centrifugation (11,270×*g*, 60 min) and lyophilization. The DOX and CUR content were determined using UV spectrophotometry at 504 nm and HPLC (Guo et al., 2017), respectively. The equations were as follows:

(1) $$\mathrm{EE}\%=\frac{\text{weight of drug in nanoparticles}}{\text{weight of feed drug}}\times 100$$

(2) $$\mathrm{DL}\%=\frac{\text{weight of drug in nanoparticles}}{\text{weight of nanoparticles}}\times 100$$

#### 2.2.5. Drug release study *in vitro*

The cumulative release of DOX and CUR from the (DOX + CUR)-FA-NPs was measured using dialysis in a release medium consisting of PBS (pH 7.4 or 6.8) with 0.5% (w/w) Tween 80 (Wang et al., 2012). In brief, 1 mL of the (DOX + CUR)-FA-NP suspension was placed in dialysis bags (MW 14,000 Da). The dialysis bags were suspended in tubes containing 100 mL of the release medium in a shaking water bath at 120 rpm and 37 °C. At each checkpoint, 25 mL of the release medium was drawn and replaced with the same volume of fresh release medium. The amounts of DOX and CUR in the release medium were calculated using the following equation:

(3) $$\text{Cumulative release\%}=\frac{C_{n}\times V+(C_{1}+C_{2}+C_{3}\ldots C_{n-1})\times V_{0}}{\text{the weight of drug in nanoparticle}}\times 100\;$$

where *C*_1–_*_n_* is the drug content in the release medium at each selected time, *V* is the total volume of the releasing medium, and *V*_0_ is the sample volume (25 mL).

#### 2.2.6. *In vitro* cytotoxicity and pharmacodynamics assay

The cytotoxicity of the blank-NPs and blank- FA-NPs was tested using the L929 cells and the MTT assay, while the *in vitro* pharmacodynamics of the different samples were evaluated using MCF-7 and MCF-7/ADR cells. One hundred microliters of blank FA-NP or blank-NP suspension at different concentrations were co-cultured with L929 cells (5 × 10^3^ cells per well) for 24 h at 37 °C and 5% CO_2_. MCF-7 and MCF-7/ADR cells, seeded at 5 × 10^3^ cells per well, were treated with 100 μL of DOX, CUR, (DOX + CUR), (DOX + CUR)-NPs, and (DOX + CUR)-FA-NPs at different concentrations as the sample groups were added and co-cultured for 24 h. All samples were prepared in triplicate. Subsequently, the medium was replaced with the same volume of PBS containing 10% MTT, and the cells were further incubated at 37 °C for 4 h. Thereafter, the medium in the wells was removed and replaced with 150 μL of DMSO. Absorbance was measured at 490 nm, and the equation used for cell viability (%) was as follows:

(4) $$\text{Cell viability\%}=\frac{\text{OD test}}{\text{OD control}}\times \;100$$

where medium only was the negative control group (100% cell viability); OD_test_ and OD_control_ are the absorbance values of the test and negative control groups, respectively.

The IC_50_ values of the different samples were calculated using SPSS (Chicago, IL). Additionally, MDR and its remission degree were characterized by the resistance index (RI) and reversal factor (RF), respectively, and were calculated as follows:

(5) $$\mathrm{RI}=\frac{{\mathrm{IC}}_{50}(A)}{{\mathrm{IC}}_{50}(B)}$$

(6) $$\mathrm{RF}=\frac{{\mathrm{IC}}_{50}(C)}{{\mathrm{IC}}_{50}(D)}$$

where IC_50_(A) is the IC_50_ of one sample on MCF-7/ADR cells, IC_50_(B) is the IC_50_ of one sample on MCF-7 cells, IC_50_(C) is the IC_50_ of DOX on MCF-7/ADR cells, and IC_50_(D) is the IC_50_ of one sample on MCF-7 cells.

#### 2.2.7. Cellular uptake

MCF-7 and MCF-7/ADR cells were seeded in a six-well plate (10^6^ cells per well). Then, 2 mL samples of (DOX, CUR, (DOX + CUR), (DOX + CUR)-NPs, and (DOX + CUR)-FA-NPs) with different concentrations of DOX and CUR at 20 μg/mL and 24 μg/mL, respectively, were added and co-cultured for 0.5, 1, and 4 h. Later, the cells were washed three times with PBS solution, and flow cytometry was used to evaluate the efficiency of cellular uptake.

#### 2.2.8. Animal studies

BALB/c nude female mice (15–20 g) were purchased from the Zhejiang Academy of Medical Sciences (Hangzhou, China). Approximately, 1 × 10^7^ MCF-7/ADR cells (0.2 mL) were subcutaneously injected into the left forelimb of the nude mice, while the tumor volume reached 50–80 mm^3^, *in vivo* biodistribution and pharmacodynamic studies were performed.

#### 2.2.9. *In vivo* distribution

Injections of 0.2 mL of (DOX + CUR), (DOX + CUR)-NPs, and (DOX + CUR)-FA-NPs with doses of DOX and CUR at 5 mg/kg and 6 mg/kg were made into the tumor-bearing nude mice via the tail vein. At each checkpoint, the mice were anesthetized, and drug distribution was analyzed using a small animal imager at excitation and emission wavelengths of 480 nm and 520 nm, respectively. The nude mice were sacrificed 8 h after injection, and the heart, liver, spleen, lungs, kidneys, and tumors were harvested. The fluorescence intensity of each organ was measured using the same method.

#### 2.2.10. Anticancer efficacy *in vivo*

*In vivo* pharmacodynamics studies lasted for two weeks. Tumor-bearing nude mice were randomly divided into five groups (*n* = 3/group). Each group was treated with (DOX + CUR)-FA-NPs, (DOX + CUR)-NPs, (DOX + CUR), or DOX containing 5 mg/kg DOX and physiological saline (negative control), respectively. Samples containing 5 mg/kg DOX (0.2 mL) were administrated every other day by tail vein injection. The tumor volume (*V*=*ab*^2^/2, *a*: the maximum length of the transplanted tumor, *b*: the maximum transverse diameter of the transplanted tumor) and the body weight of the nude mice were measured throughout the experiment.

#### 2.2.11. Statistical analysis

The experimental results were expressed as the mean ± standard error. The *t*-test was used for the statistical analysis. Statistical significance was set at *p*<.05.

## 3. Results and discussion

### 3.1. Pre-polymer characterization

**Figure 3.**
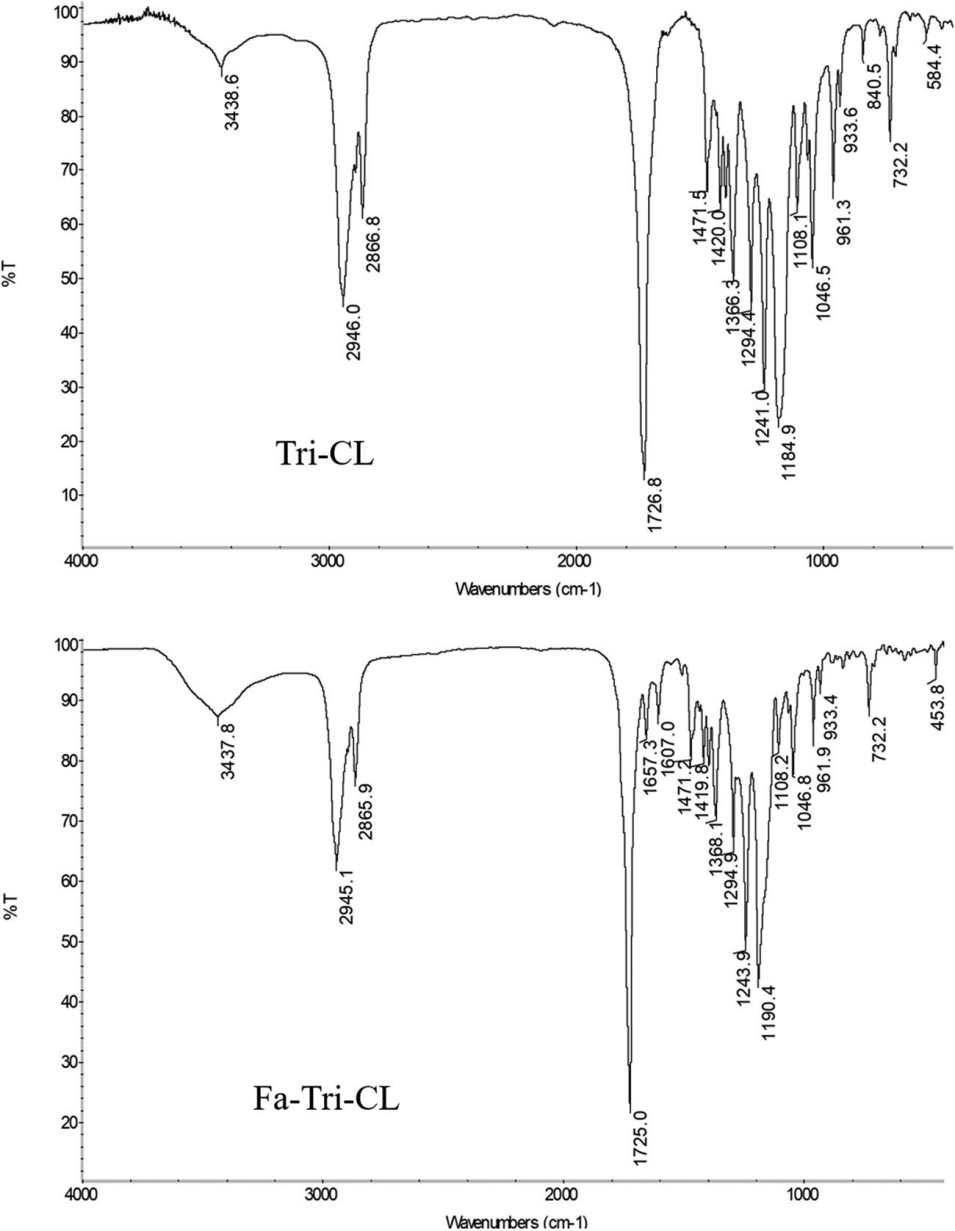
FT-IR spectra of TRI-PCL and FA-TRI-PCL.

**Figure 4.**
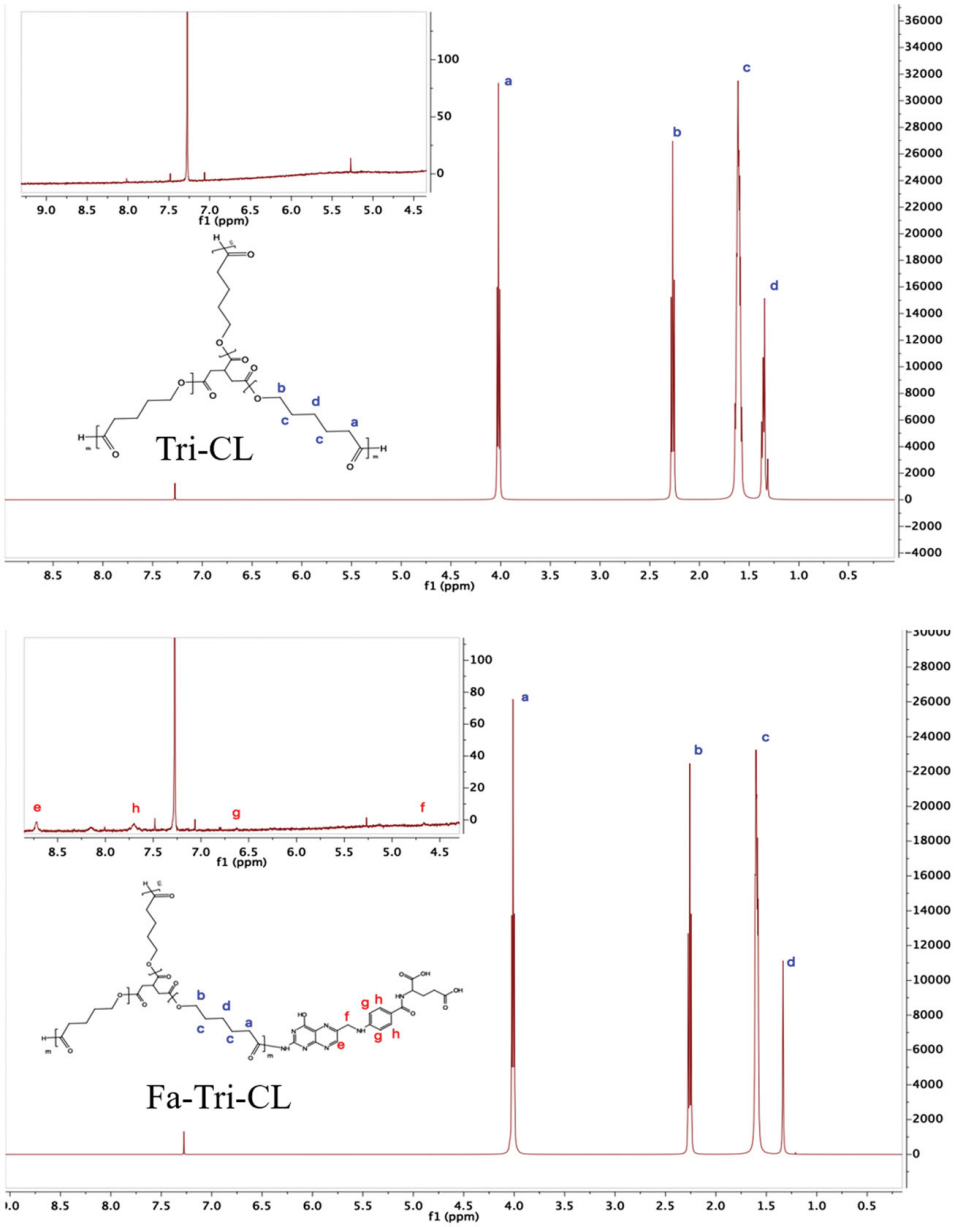
^1^H NMR spectra of TRI-PCL and FA-TRI-PCL.

The FT-IR spectra of TRI-CL and FA-TRI-CL are shown in Figure 3. For TRI-CL, the stretching vibrational peak of the terminal hydroxyl group was at 3438.6 cm^−1^; the peaks at 2946.0, 2866.8, 1471.5, and 732.2 cm^−1^ were attributed to C–H bonds in the methylene group; the peaks at 1726.8 cm^−1^, and 1184.9 and 1108.1 cm^−1^ were characteristic of the C–O and C–O–C bonds in the ester group. Compared with the spectrum of TRI-CL, the IR spectrum of FA-TRI-CL showed additional peaks at 1657.3, 1512.9, and 1471.2 cm^−1^, which corresponded to the C–H bond in the benzene ring of FA. The results of ^1^H NMR spectra are shown in Figure 4. For TRI-CL, the chemical shift of the methylene proton in the TRI backbone was at 2.35 ppm; the chemical shift of the methylene protons in the CL repeating units was observed at 4.07 (a), 2.32 (b), 1.65 (c), and 1.40 (d) ppm. For FA-TRI-CL, the peak at 8.71 ppm (e) was attributed to the last methyl hydrogen atom of the pterin ring in FA; the peaks at 7.62 ppm (h) and 6.61 ppm (g) were the C–H of the benzene ring in FA; and the peak at 4.69 ppm (f) was attributed to methylene (Anirudhan et al., 2017). In summary, the FA-TRI-CL was successfully synthesized.

### 3.2. Characterization of NPs

**Figure 5.**
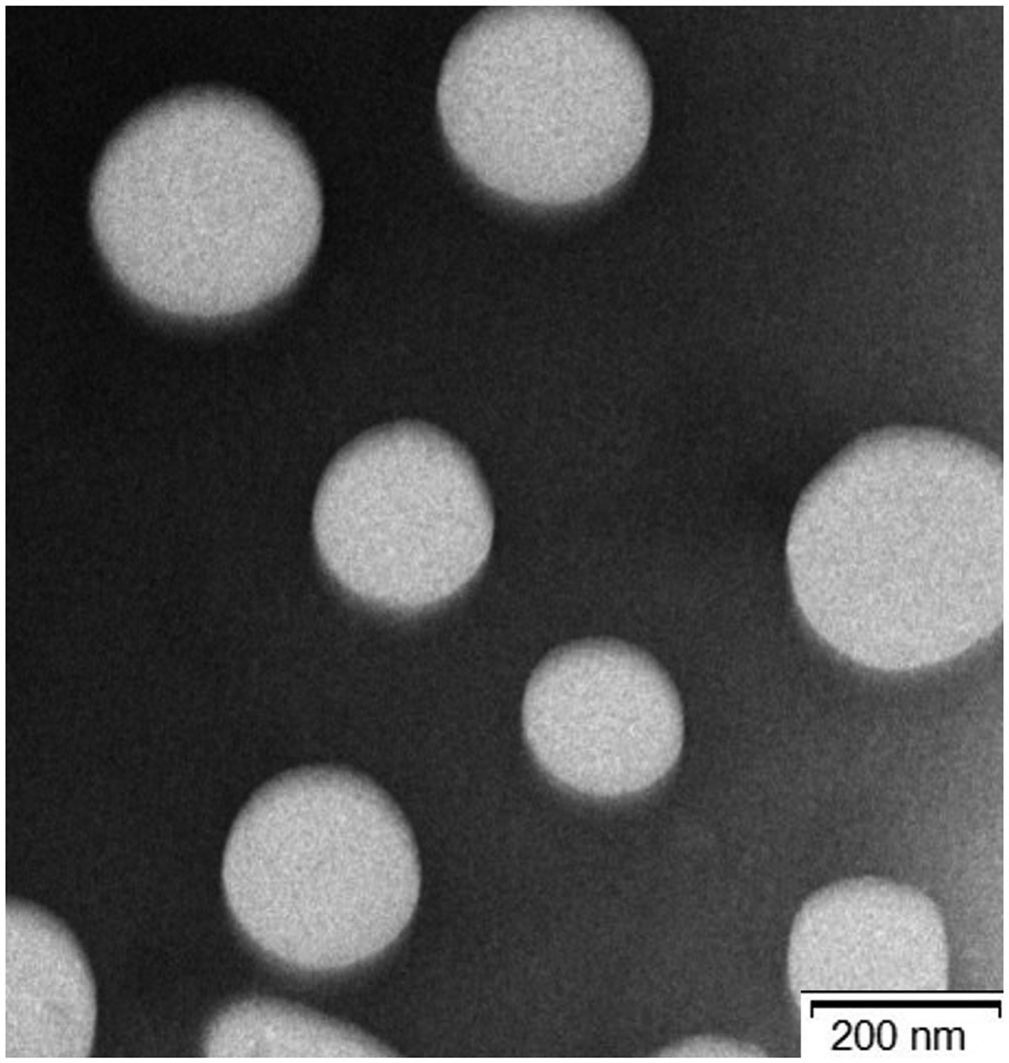
TEM photograph of (DOX + CUR)-FA-NPs (×15,000 magnification).

**Figure 6.**
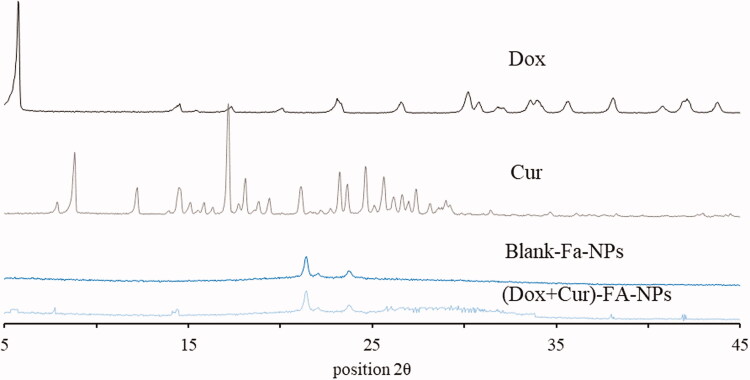
XRD curves of DOX, CUR, blank-FA-NPs, and (DOX + CUR)-FA-NPs.

As illustrated in Table 1, the particle sizes were 161.51 ± 1.70, 170.46 ± 2.57, 183.48 ± 0.82, and 186.53 ± 2.78 nm for the blank-NPs, blank-FA-NPs, (CUR + DOX)-NPs, and (CUR + DOX)-FA-NPs, respectively, with PDIs of less than 0.1, indicating that the nanoparticles were well distributed. The surface zeta-potential of the prepared nanoparticles was electronegative in the range of −8.41 ± 0.87 to −18.87 ± 1.13 mV, resulting from a carboxyl group. By comparison, owing to two more carboxyl groups in FA, the FA-modified nano-carriers showed a lower zeta-potential, which contributed to increased stability in the delivery system (Doane et al., 2012; Shao et al., 2015). The DL and EE of the CUR and DOX in the delivery systems were both higher than 11.29% and 77.80%, respectively. The DL values for both drugs were excellent, when compared with previous reports (Duan et al., 2012; Zhao et al., 2015; Lv et al., 2016). Additionally, compared with the other drugs, CUR had improved DL and EE values, which was attributed to the CUR having a stronger solvency in the acetone/water system. The results of the TEM and XRD are shown in Figures 5 and 6, respectively. (DOX + CUR)-FA-NPs have a regular circular shape with a particle size of approximately 180 nm (Figure 5), which is consistent with the particle size measured by the Malvern particle size analyzer (Malvern Instruments, Worcestershire, UK). DOX and CUR have numerous high intensity peaks, which disappeared in the (DOX + CUR)-FA-NPs, indicating that DOX and CUR were encapsulated in the nanoparticles rather than adsorbed on their surface (Figure 6).

**Table 1.** Characterization of different nanoparticles.

Sample	Size (nm)	PDI	Zeta (mV)	EE% CUR/DOX	DL% CUR/DOX
Blank NPs	161.5 ± 1.70	0.072 ± 0.011	–10.03 ± 1.63	–	–
Blank FA-NPs	170.4 ± 2.57	0.054 ± 0.009	–16.01 ± 0.31	–	–
(CUR + DOX)-NPS	183.4 ± 0.82	0.042 ± 0.018	–8.41 ± 0.87	97.41/77.80	19.54/12.45
(CUR + DOX)-FA-NPS	186.5 ± 2.78	0.024 ± 0.008	–18.87 ± 1.13	97.64/78.13	20.27/11.29

### 3.3. Drug release study *in vitro*

**Figure 7.**
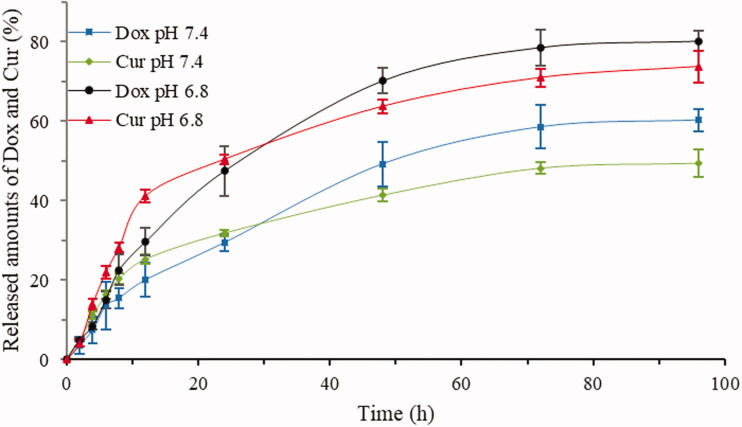
Release curves of (DOX + CUR)-FA-NPs at different pH values.

To understand the pharmacokinetics in different physiological environments, the drug release behavior *in vitro* was carried out at 37 °C under simulative microenvironments for blood (pH = 7.4) and tumor (pH = 6.8; Gao & Lo, 2018). The results are listed in Figure 7. Within 96 h, the cumulative release rates of DOX (80.2%) and CUR (73.7%) in the (CUR + DOX)-FA-NPs at pH 6.8 were significantly greater than those of DOX (60.3%) and CUR (49.4%) at pH = 7.4, indicating that weaker side effects could be achieved. Additionally, when CUR and DOX were compared, and the release rate of CUR (41.2%/21.3%, pH 6.8/pH 7.4) was faster than that of DOX (29.7%/20.1%, pH 6.8/pH 7.4) at 12 h, but the regulation was reversed after 24 h. In this release mechanism, the CUR interfered with the tumors and organs faster than that of the DOX in the previous period, which not only reduced the toxicity of the DOX in the normal tissues, but also inhibited in advance the P-gp in the tumors.

### 3.4. *In vitro* cytotoxicity and pharmacodynamics assay

**Figure 8.**
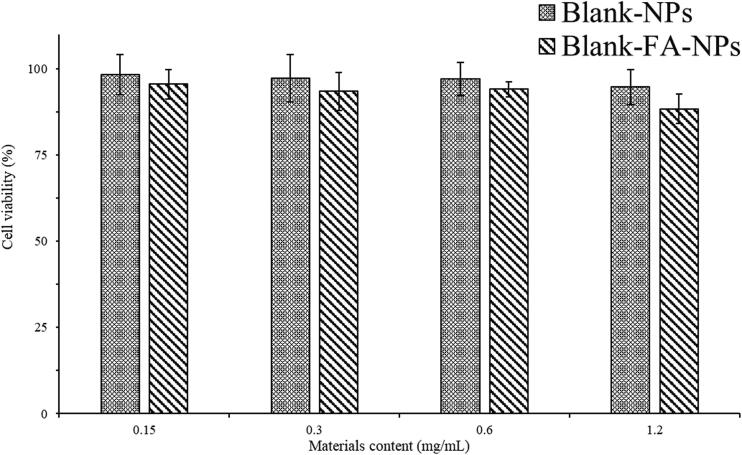
Cytotoxicity of the materials in the L929 cells.

**Figure 9.**
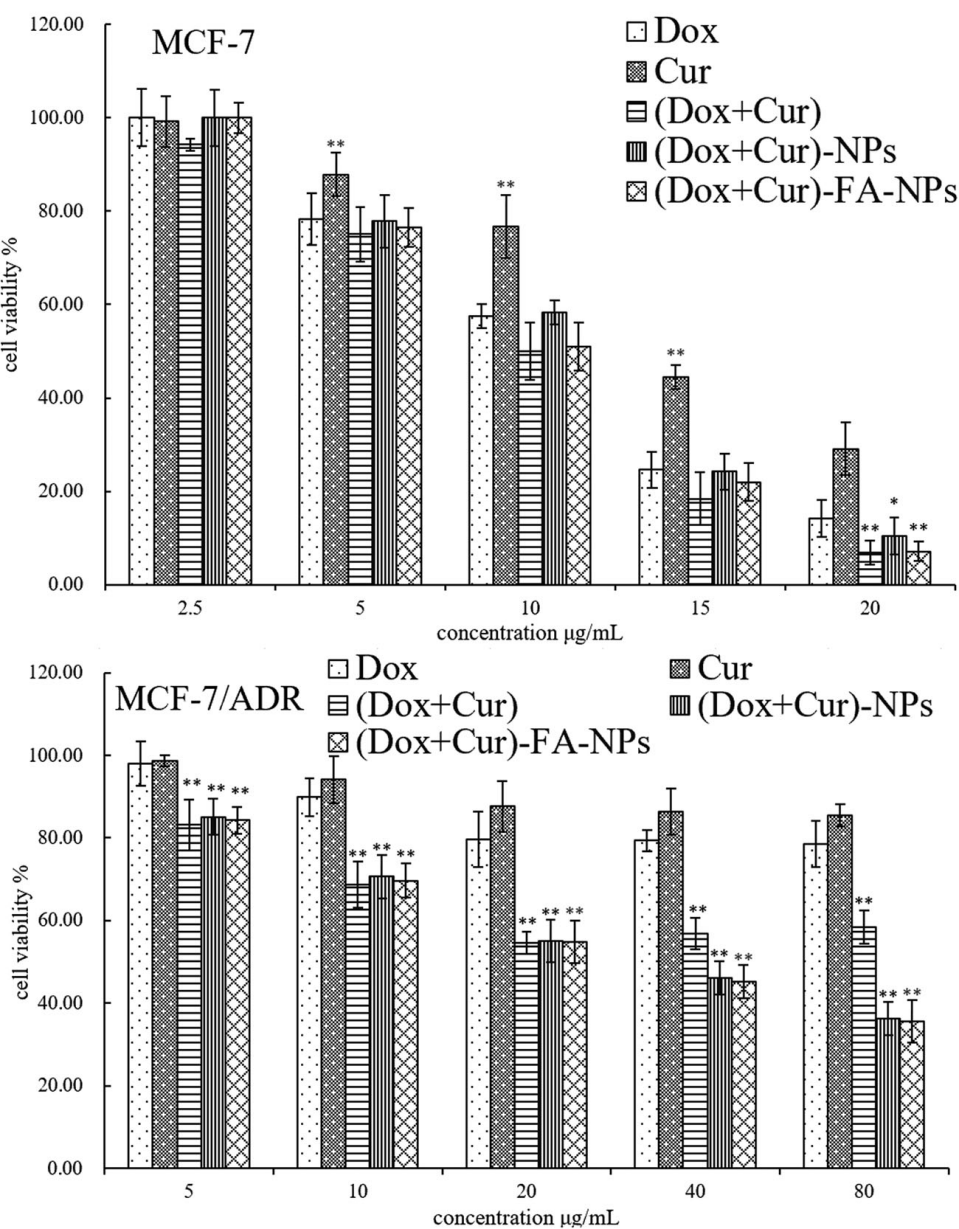
The antiproliferation effects of DOX, CUR, (DOX + CUR), (DOX + CUR)-NPs, and (DOX + CUR)-FA-NPs in the MCF-7 and MCF-7/ADR cells.

The results of the cytotoxicity study for the materials are shown in Figure 8. With the increasing concentration of the blank-NPs and blank-FA-NPs (0.15–1.2 mg/mL), the L929 cell viability for each group decreased very slightly. Cell viability was above 88%, indicating that the materials’ cytotoxicity for both nanoparticle carriers had good biocompatibility and low cell toxicity. The anti-proliferative effects of each drug-loaded sample group against the MCF-7 and MCF-7/ADR cells are summarized in Figure 9, where the IC_50_, RI, and RF values of each sample with the test cells were calculated using SPSS (Chicago, IL), and the results are listed in Tables 2 and 3, respectively. All drug-loaded carriers displayed dose-dependent anticancer activity against both test cells (Figure 9). Cell viability ranged from 99.45% to 10.88% after 24 h of co-incubation with the different sample groups. For the MCF-7 cells, the anti-proliferative ability decreased in the following order: (CUR + DOX)>(CUR + DOX)-FA-NPs>(CUR + DOX)-NPs > DOX > CUR. In comparison with each sample group, the sample groups with combination drug therapy ((CUR + DOX), (CUR + DOX)-FA-NPs, and (CUR + DOX)-NPs) displayed stronger anti-cell proliferation than those with a single drug (CUR or DOX). This demonstrated that the CUR and DOX combination is an effective strategy for breast cancer treatment. (CUR + DOX) displayed the best anticancer activity, as the cells were in direct contact with the drugs, and both lipophilic drugs easily penetrated the cell membranes. On the other hand, when CUR and DOX were loaded into nanocarriers, cell cytotoxicity was significantly reduced. The different anti-proliferative abilities of (CUR + DOX)-FA-NPs and (CUR + DOX)-NPs were mainly determined from the cellular uptake of each sample. For MCF-7/ADR cells, combination therapy with multiple drugs had better pharmacodynamics. When the DOX concentration was 5–20 μg/mL, a similar regulation of anti-proliferative ability among all samples was observed. As the DOX concentration increased (40–80 μg/mL), (DOX + CUR)-FA-NPs and (DOX + CUR)-NPs showed better anti-proliferative abilities than (DOX + CUR). In this phase, as the drug content increased, more (DOX + CUR)-loaded nanocarriers were taken up into the cells because of an increased cell-uptake efficiency (Guo et al., 2019).

**Table 2.** The IC_50_ value of Dox, Cur, (Cur + Dox), (Cur + Dox)-NPs, and (Cur + Dox)-FA-NPs in MCF-7 cells and MCF-7/ADR cells.

Samples	MCF-7	MCF-7/ADR
	IC_50_ (µg/mL)	IC_50_ (µg/mL)
Dox	12.13	159.40
Cur	15.56	197.19
(Dox + Cur)	10.65	93.55
(Dox + Cur)-NPs	11.46	45.36
(Dox + Cur)-FA-NPs	11.15	43.74

**Table 3.** Resistance index and resistance reversal factor.

Samples	RI	RF
Dox	13.14	1.00
(Dox + Cur)	10.65	1.70
(Dox + Cur)-NPs	3.96	3.51
(Dox + Cur)-FA-NPs	3.92	3.64

In comparison with MCF-7 and MCF-7/ADR cells, MCF-7/ADR cells showed better cell viability than MCF-7 cells after co-culture with the sample groups (Figure 9). Meanwhile, the IC_50_ value of each sample group against the MCF-7/ADR cells (43.74–197.19 μg/mL) was greater than that of the MCF-7 (10.65–15.56 μg/mL; Table 2). The above results demonstrated that MCF-7/ADR cells had higher MDR than MCF-7 cells. Additionally, the RI and RF values of the DOX, (DOX + CUR), (CUR + DOX)-NPs, and (DOX + CUR)-FA-NPs were 13.14/1.00, 10.65/1.70, 3.96/3.51, and 3.92/3.64, respectively (Table 2). The highest RI value of the DOX group showed the strongest MDR against MCF-7/ADR cells. When CUR and DOX were co-delivered, the RI values decreased, and the RF values increased, demonstrating that CUR could improve the resistance of DOX against the MCF-7/ADR cells. Furthermore, when CUR and DOX were co-delivered by nanocarriers, the variation in the RI and RF values was more prominent than that of the (CUR + DOX) group, indicating that a single drug mixture could more easily efflux from the cell via the P-gp than the drug-loaded nanoparticles.

### 3.5. Cellular uptake study

**Figure 10.**
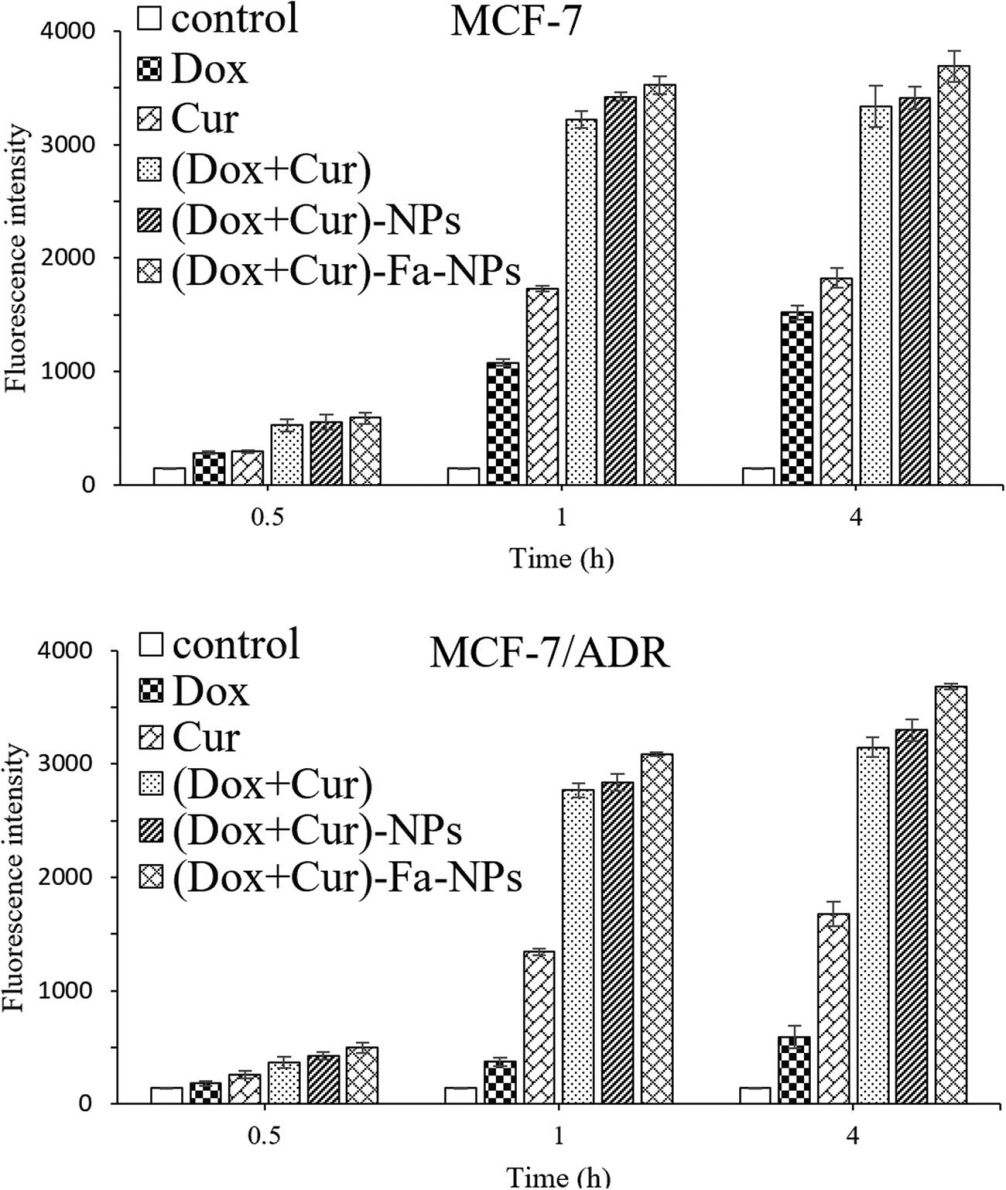
Flow cytometry analysis of the cellular uptake.

The effects of the MDR on cellular uptake and the combination therapy on improving MDR were verified. A cellular uptake study was performed, and the results are shown in Figure 10. The mean fluorescence intensity (MFI) showed that the uptake of all drug formulations increased with time in both MCF-7/ADR and MCF-7 cells. By comparison, the MFI of the DOX group in the MCF-7 cells was much higher than that in the MCF-7/ADR (0.5 h, 1.54 times; 1 h, 2.89 times; 4 h, 2.57 times, respectively). As the intracellular concentration of the DOX increased, the efflux mechanism was much stronger in the MCF-7/ADR cells than in the MCF-7 cells, inducing a decrease in the concentration of DOX. For the CUR, the MFI values between the cells were similar, indicating that it partially inhibited the efflux mechanisms in the cells (especially MCF-7/ADR). Furthermore, at each checkpoint, the ratio of MFI_(DOX + CUR)_/(MFI_DOX_+MFI_CUR_), MFI_(CUR + DOX)-FA-NPs_/(MFI_DOX_+MFI_CUR_), and MFI_(CUR + DOX)-NPs_/(MFI_DOX_+MFI_CUR_) was between 1.2 and 2.1 (>1) in the MCF-7/ADR cells, indicating that the co-delivery of CUR and DOX could effectively improve the efflux of the DOX, and is an effective strategy for combination oncology therapies. The above results were also confirmed the results from the analysis of cell anti-proliferation experiments.

### 3.6. Tumor targeting *in vivo*

**Figure 11.**
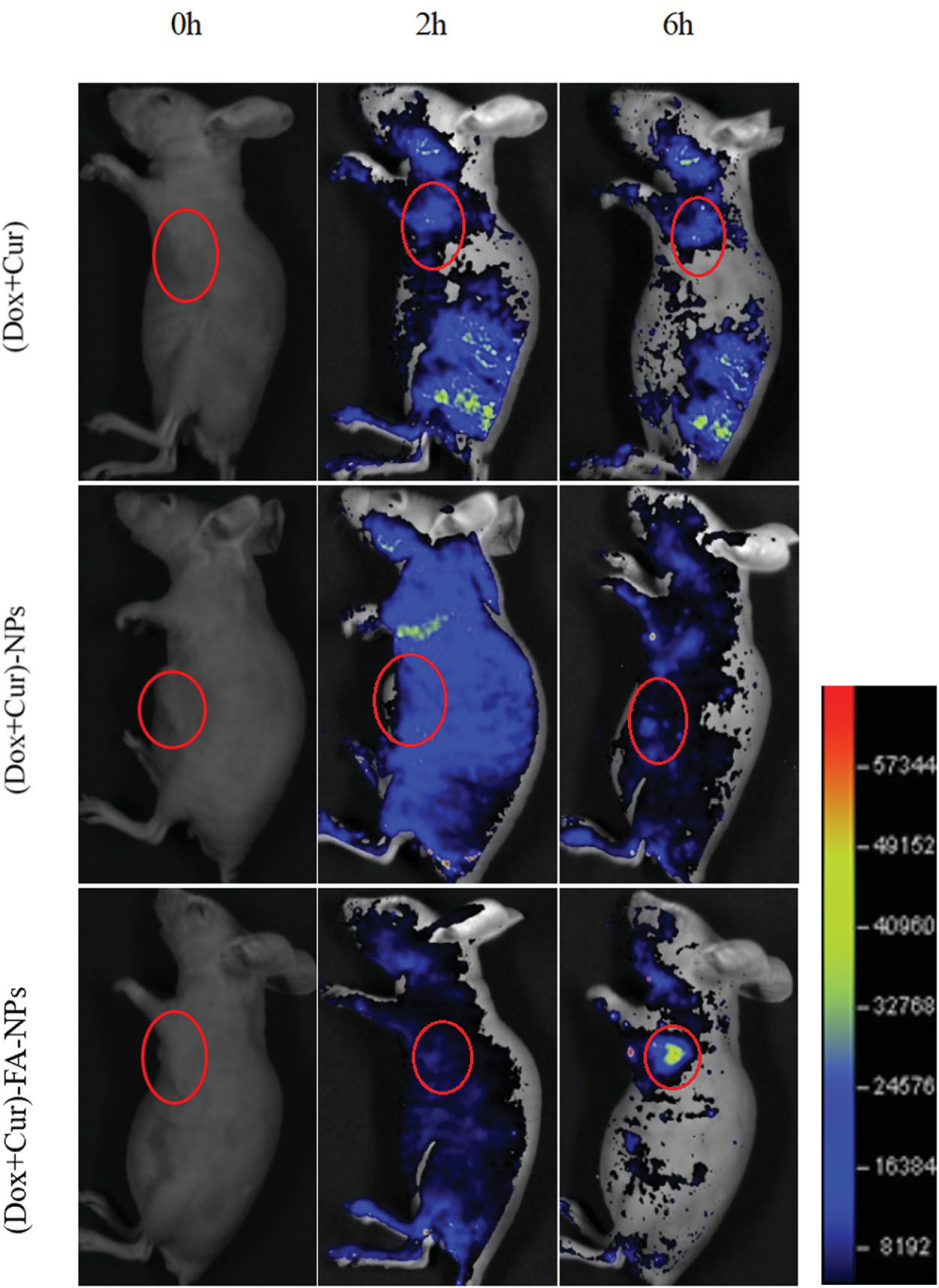
Biodistribution imaging of the whole body after IV injections with different drug formulations.

**Figure 12.**
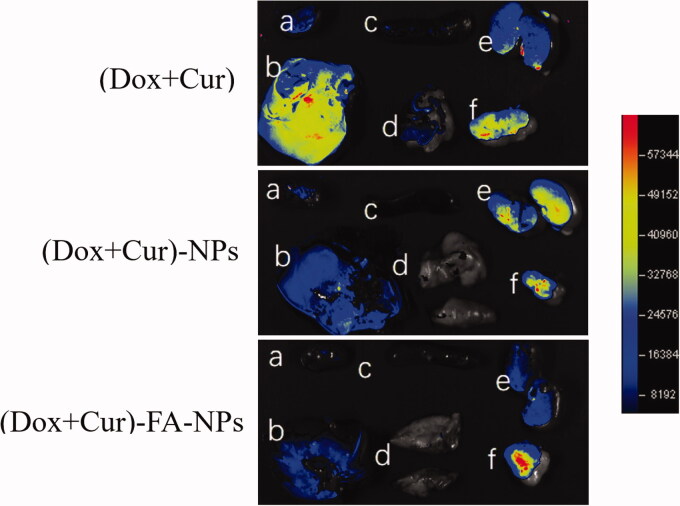
*In vivo* distribution of (DOX + CUR), (DOX + CUR)-NPs, and (DOX + CUR)-FA-NPs in the tissues. (a) Heart; (b) liver; (c) spleen; (d) lung; (e) kidney; (f) tumor.

The potential targeted distribution of (DOX + CUR), (DOX + CUR)-NPs, and (DOX + CUR)-FA-NPs in the MCF-7/ADR tumor-bearing nude mice was evaluated by intravital fluorescent imaging analysis. The results are shown in Figures 11 and 12, respectively. No obvious tissue targeting was observed in either the (DOX + CUR) or (DOX + CUR)-NPs after administration through the tail vein (Figure 11). For (DOX + CUR)-FA-NPs, there was a significant fluorescence aggregation at the tumor site at 6 h, indicating that (DOX + CUR)-FA-NPs had a good targeting selectivity for MCF-7/ADR, due to the specific docking between FA and the FA receptor (from tumor). Moreover, the *ex vivo* tissue images 8 h after administration are shown in Figure 12. In the (DOX + CUR) group, the drugs were mainly distributed in the liver, kidneys, and tumors. Meanwhile, slight fluorescence intensities were still measured in the heart, spleen, and lungs. The above results demonstrated that (DOX + CUR) had no tissue targeting and could induce toxicity in the heart, liver, and kidneys. For (DOX + CUR)-NPs, the fluorescence intensity in the heart and liver was obviously decreased when compared to that of (DOX + CUR), and the drugs were mainly distributed in the kidneys and tumors, while for (DOX + CUR)-FA-NPs, the drugs were mainly distributed in the tumor. In comparison to (DOX + CUR)-NPs, the fluorescence intensity in the kidneys decreased significantly, while the fluorescence intensity in the heart and liver was further decreased. These results demonstrated that the (DOX + CUR)-FA-NPs had excellent tumor-targeting selectivity and biosecurity.

### 3.7. Anticancer efficacy *in vivo*

**Figure 13.**
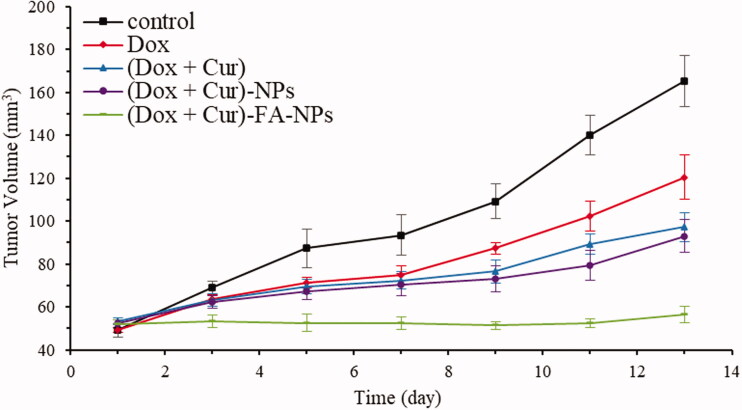
MCF-7/ADR tumor growth curves for the different drug formulations after the treatments.

**Figure 14.**
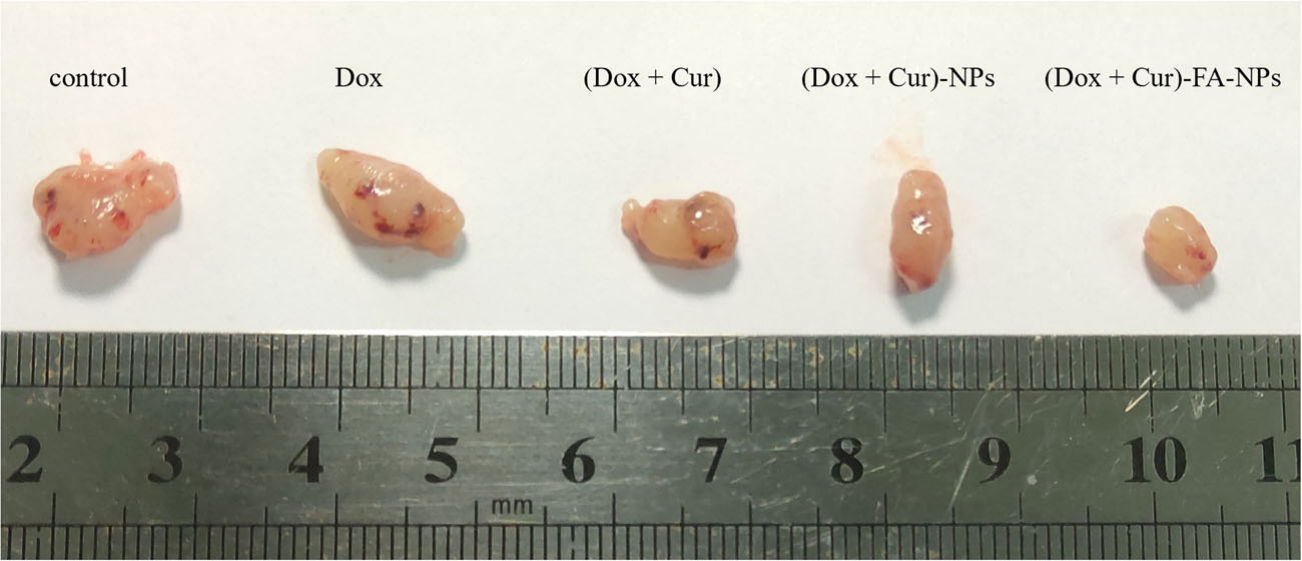
Photos of the tumors after various treatments.

**Figure 15.**
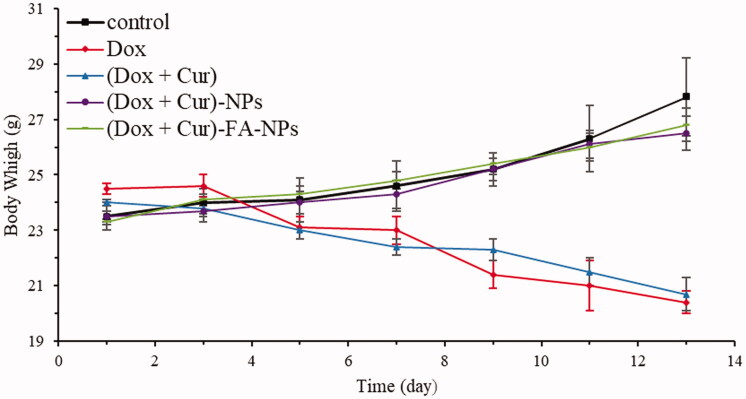
Body weights of the mice after treatments with various drug formulations.

To study the therapeutic effects of (DOX + CUR)-FA-NPs *in vivo*, different drug formulations were administered to tumor-bearing nude mice. The variations in tumor volume and body weight are displayed in Figures 13–15. Only the (DOX + CUR)-FA-NPs group successfully inhibited tumor growth, while the tumor volume continued to increase after treatment with the other formulations (Figure 13). The post-anatomical view of the tumor at the end of the experiment is shown in Figure 14; the tumor areas with physiological saline, DOX, (DOX + CUR), (DOX + CUR)-NPs, and (DOX + CUR)-FA-NPs treatments were 165 ± 12 cm^3^, 120 ± 10 cm^3^, 97 ± 7 cm^3^, 93 ± 8 cm^3^, and 56 ± 4 cm^3^, respectively. In comparison with the tumor volume (physiological saline group as the baseline), the final tumor growth inhibition of DOX, (DOX + CUR), (DOX + CUR)-NPs, and (DOX + CUR)-FA-NPs was 27.2%, 41.2%, 43.8%, and 65.8%, respectively. The results further demonstrated that (DOX + CUR)-FA-NPs had the best therapeutic effect due to their pharmacodynamics, and pharmacokinetics *in vivo*.

Additionally, the variation in the mice body weights could reflect the biosafety of the dosage form *in vivo*. The mice continued to show weight loss after treatment with DOX and (DOX + CUR), while for physiological saline, (DOX + CUR)-NPs, and (DOX + CUR)-FA-NPs, the body weights showed stable growth (Figure 15). The above results indicate that direct drug administration (via i.v.) could induce serious systemic toxicity, while the nano-delivery systems improved biocompatibility, which aligns with the results of cytotoxicity and pharmacodynamics *in vivo*. Further comparison between DOX and (DOX + CUR) showed that the percentage of weight loss was 18.7% and 13.7% during the treatment, indicating that the combination of CUR and DOX could reduce the toxicity and side effects of the DOX.

## 4. Conclusions

In conclusion, to improve the MDR, toxicity, and target selection of the DOX, and to enhance its clinical efficacy, a strategy combining nano-delivery systems with combination drug therapy was proposed. In this study, CUR was selected as the other model drug due to its low toxicity, protective effects on DOX toxicity, and P-gp inhibition. First, we designed and synthesized FA-TRI-CL as the pro-polymer of the nanocarrier, in which the FA block provided the target docking and the star polyester improved the DL values. The new (DOX + CUR)-FA-NPs with ideal diameter and size distribution, and good EE and DL values were prepared using the emulsion solvent evaporation method. Second, the (DOX + CUR)-FA-NPs exhibited a gradual drug release process. Meanwhile, *in vitro* cytotoxicity and pharmacodynamics studies and cellular uptake tests demonstrated that (DOX + CUR)-FA-NPs have good biocompatibility and the best anti-proliferative and cellular uptake abilities. Moreover, improved resistance of DOX due to the CUR was also verified. Finally, *in vivo* pharmacokinetics and pharmacodynamics showed that (DOX + CUR)-FA-NPs not only have obvious tumor targeting, but also reduce drug accumulations in important organs, and displayed the best anticancer efficacy. All the above results demonstrate that this could be a promising system for future clinical oncology therapies.
